# Sweet Syndrome, Not so Sweet during an Ulcerative Colitis Flare Especially When You Cannot Eat

**DOI:** 10.1155/2021/9940391

**Published:** 2021-09-30

**Authors:** Courtney Stead, Shahrad Hakimian, Christina Luffman, Zendee Elaba, Gregory Orlowski, Neil Marya

**Affiliations:** ^1^Department of Medicine, University of Massachusetts Medical School, 55 Lake Avenue North, Worcester, MA 01655, USA; ^2^Division of Digestive Diseases, University of California Health, Los Angeles, CA, USA; ^3^Department of Pathology, University of Massachusetts Medical School, Worcester, MA, USA; ^4^Department of Dermatology, University of Massachusetts Medical School, Worcester, MA, USA; ^5^Division of Gastroenterology, University of Massachusetts Medical School, Worcester, MA, USA

## Abstract

Sweet syndrome is a rare skin condition characterized by painful papules, nodules, or plaques with dense neutrophilic infiltrate in the upper dermis. It has been observed as idiopathic (classical), malignancy-associated, and drug-induced. The pathogenesis is not completely understood, but it is thought to involve hypersensitivity reactions to specific triggers. In some cases the etiology is unclear or may be multifactorial. We present a case of Sweet syndrome secondary to ulcerative colitis flare versus adalimumab re-induction.

## 1. Introduction

Sweet syndrome, or acute neutrophilic dermatosis, is a rare skin condition characterized by painful papules, nodules, or plaques with dense neutrophilic infiltrate in the upper dermis [1]. It is often accompanied by fever and peripheral neutrophilia. Less common, but reported, complications include arthralgias, myalgias, nausea, vomiting, diarrhea, headache, and conjunctivitis. This syndrome is more frequently seen in women, most prevalent in adults aged 30–70 years old, but has also been reported in children [2]. The pathogenesis of the disease remains unknown; however, it has been postulated that it may be a hypersensitivity reaction given its relation to autoimmune diseases, malignancies, and infections. Here, we report a case of Sweet syndrome in a patient with pan-ulcerative colitis recently re-induced with adalimumab.

## 2. Case Report

**Figure 1:**
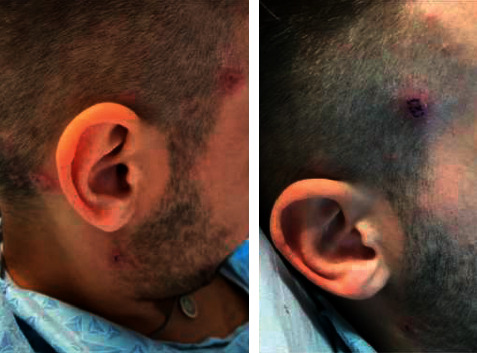
Firm, erythematous, and umbilicated papules with central erosion and crust. The lesions were observed on the face, scalp, and jaw.

**Figure 2:**
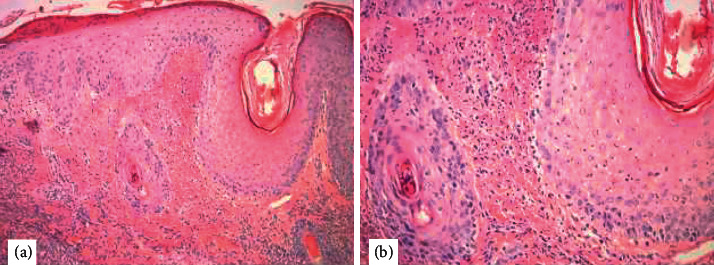
Histologic sections show subcorneal pustules and dense neutrophilic dermal infiltrate ((a)-4X and (b)-10X). Special stains including gram, AFB, and FITE were negative for definitive microorganisms.

A 22-year-old male with a history of pan-ulcerative colitis (UC) recently re-induced on adalimumab presented to the hospital with odynophagia and inability to swallow secondary to significant oral ulceration and 20–25lb weight loss. He was diagnosed with UC at 20 years old and was treated with a course of prednisone along with induction with adalimumab. He remained asymptomatic on adalimumab for 8 months until he lost his insurance and self-discontinued the medication. He remained minimally symptomatic for 2 years and was lost to follow-up. However, he finally presented to clinic with one month of bloody diarrhea and weight loss and was eventually re-induced with adalimumab and a prednisone taper after being diagnosed with a UC flare. His gastrointestinal symptoms resolved quickly; however, he developed severe progression of oral mucosal ulcerations and a papular rash approximately 7–10 days after adalimumab re-induction. Due to worsening odynophagia, he presented to the hospital with severe dehydration. On admission, the patient was afebrile with laboratory investigations significant for C-reactive protein (CRP) 151.5, white blood cell count (WBC) 13.0, and neutrophils 10.18 (78.4%). Otolaryngology was consulted to perform laryngoscopy, which was significant for ulcerations along the epiglottis. Biopsy of an ulceration was collected at that time. Endoscopy was not performed. On day two of hospitalization, he developed a fever to 38.3 C. At this time, the patient was noted to have erythematous, scaled papules, and nodules on his face, head, trunk, and buttocks that drained transparent, yellow discharge (Figure 1). Dermatology was consulted and performed a punch biopsy of the skin lesions and oral ulcers. The gingival biopsies were nonspecific; however, skin biopsy was significant for neutrophilic infiltrate in the dermis with formation of microabscesses within the epidermis and superficial dermis (Figure 2). The constellation of the patient's presenting symptoms, biopsy findings, and disease context (i.e., onset of symptoms during a UC flare two days following adalimumab induction) raised suspicion for drug-induced vs. classic Sweet Syndrome. Thus, adalimumab was discontinued, and he was switched to infliximab therapy. In addition to this management, the patient's prednisone dose was increased. At a dermatology follow-up appointment 11 days later, the patient's skin lesions were nearly resolved, and he no longer had oral ulcers.

## 3. Discussion

There are three clinical types of Sweet syndrome: idiopathic (classic), malignancy-associated, and drug-induced. Different diagnostic criteria have been established for each type. In the classic type, lesions may resolve after weeks to months untreated. In the malignant-associated type, symptoms may resolve following remission of the cancer. In drug-induced type, improvement may be seen after stopping the associated medication. Regardless of the subtype, systemic corticosteroids are the first-line treatment, and resolution of symptoms may begin as soon as 72 hours after initiation of therapy. In this case, the onset of skin lesions consistent with Sweet syndrome during an ulcerative colitis flare, two days post re-induction of adalimumab raises the questions of classical versus drug-induced Sweet syndrome. A 2007 review of acute neutrophilic dermatosis found that classic Sweet syndrome is often associated with upper respiratory or gastrointestinal infections, as well as inflammatory diseases such as ulcerative colitis and Crohn's Disease [1]. In 2005, a review documented that 35 cases of Sweet syndrome associated with inflammatory bowel disease had been documented worldwide [3]. Drug-induced Sweet syndrome has also been well documented as associated most commonly with granulocyte colony-stimulating factor, tretinoin, sulfamethoxazole and trimethoprim, bortezomib, and azathioprine [1]. Tumor necrosis fator (TNF) inhibitors have not been commonly documented as associated with Sweet's syndrome; however, one 2015 case report suggested its association with adalimumab re-induction in Crohn's Disease. In that case, discontinuation of adalimumab prompted full recovery of the skin lesions [4]. Oral Sweet's syndrome, presenting with mouth ulcers, has also been documented in the literature [5–8]. In one report, a patient had ulcerative colitis and was taking adalimumab. This report attributed association to be to IBD, rather than adalimumab [8].

The pathogenesis of Sweet's syndrome is not well understood. It is postulated that certain drugs or diseases may stimulate cytokines that increase neutrophil activation, leading to the syndrome's manifestations of fever, neutrophilia, and neutrophil infiltration to the dermis [9, 10]. However, in these cases, it is often pro-inflammatory drugs, or drugs that are anti, pro-inflammatory cytokines, that are associated with the syndrome. Adalimumab blocks a pro-inflammatory agent, tumor necrosis factor alpha (TNF-*α*), and therefore, it is unclear what the role of this drug could be in the pathogenesis of Sweet's syndrome. Alternatively, the onset of oral ulcers and skin lesions days after re-induction of adalimumab, along with a previously documented similar case, supports an association. The lack of a development of skin lesions following multiple infliximab infusions may argue against a drug-induced etiology or may suggest that the medication-induced component of this condition is medication specific. Other than one case study where Sweet's syndrome was observed after Pegfilgrastim administration but resolved following discontinuation and transition to filgrastim [11]; there is very little literature on this topic. Lastly, given the known association with inflammatory bowel disease and this patient's coinciding flare, Sweet's syndrome secondary to a UC flare cannot be excluded. Regardless of the cause, medication or IBD itself, it is important to include this syndrome as part of the differential diagnosis of rashes, skin, and oral ulceration in presence of IBD and after administration of biologic agents such as TNF inhibitor therapy including adalimumab.

## Data Availability

Data are available from the primary author, Courtney Stead, upon request (courtney.stead@umassmemorial.org).
